# 555. Estimating Antibiotic Resistance Following Antibiotic Treatment in Outpatients: a Retrospective Study

**DOI:** 10.1093/ofid/ofaf695.164

**Published:** 2026-01-11

**Authors:** Dor Atias, Bat-Sheva Gottesman, Marcelo Low, Uri Obolski, Michal Chowers

**Affiliations:** Faculty of Medical & Health Sciences Tel-Aviv University, Tel-Aviv, Tel Aviv, Israel; Meir MC, Kfar Saba, HaMerkaz, Israel; Clalit Health Services, Tel Aviv, Tel Aviv, Israel; Faculty of Medical & Health Sciences, Tel Aviv University, Tel Aviv, Tel Aviv, Israel; Faculty of Medical & Health Sciences, Tel Aviv University., Tel Aviv, Tel Aviv, Israel

## Abstract

**Background:**

Understanding the impact of antibiotic use on the risk of future resistant infections is essential for appropriate antibiotic therapy. Current assessments are largely based on expert opinions.

**Figure 1:**
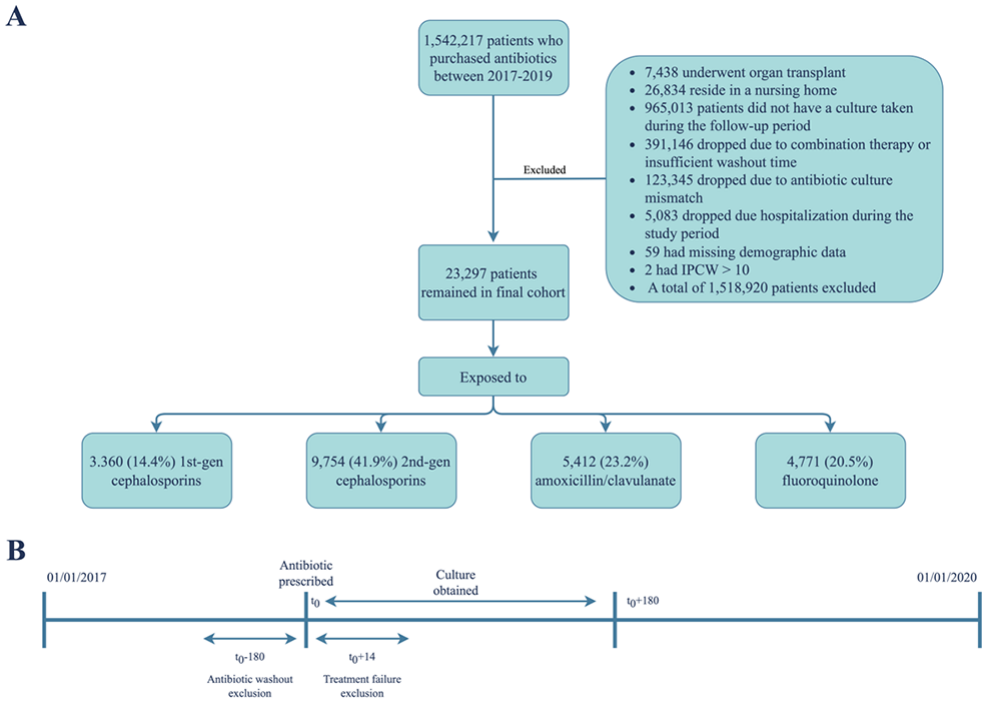
Patient included & study plan A. Flow chart describing patient selection B. Study plan

**Figure 2.**
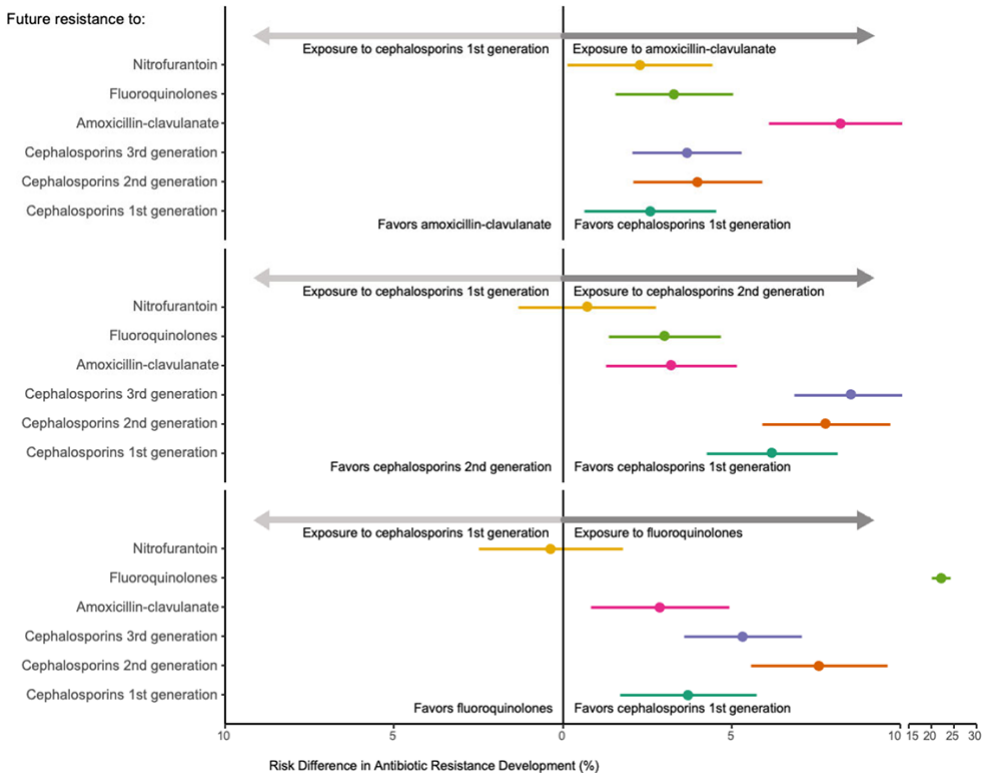
Risk Differences of Future Antibiotic Resistance When Exposed to Various Antibiotics Relative to 1st CEP. The x-axis represents the risk difference in antibiotic resistance development, with horizontal lines depicting confidence intervals. The x-axis is split between 10–15 for visualization purposes. The y-axis depicts resistance to different antibiotics: nitrofurantoin, Ciprofloxacin, AMC, third-generation cephalosporins, 2nd CEP, and 1st CEP. The upper, middle, and lower panels represent comparisons with AMC, 2nd CEP, and FQ, respectively. Positive risk differences indicate a higher risk of resistance development relative to 1st CEP.

**Methods:**

A retrospective study including adult HMO members who purchased cefazolin, cefuroxime, amoxicillin-clavulanate (AMC), or fluoroquinolones (FQ) between 1/1/2017 and 31/12/2019. (Figure 1) The outcome was the antibiotic susceptibility profile of the first urine culture within six months from antibiotic purchase. We used matching, standardization, post-matching covariate adjustments and inverse probability of censoring weighting to correct for confounding and selection bias and estimate the risk difference (RD) for resistance. The analysis emulated a trial where individuals receiving either 1st or 2nd generation cephalosporins (CEP) in the exposure group were assigned alternative antibiotics treatments.

**Figure 3:**
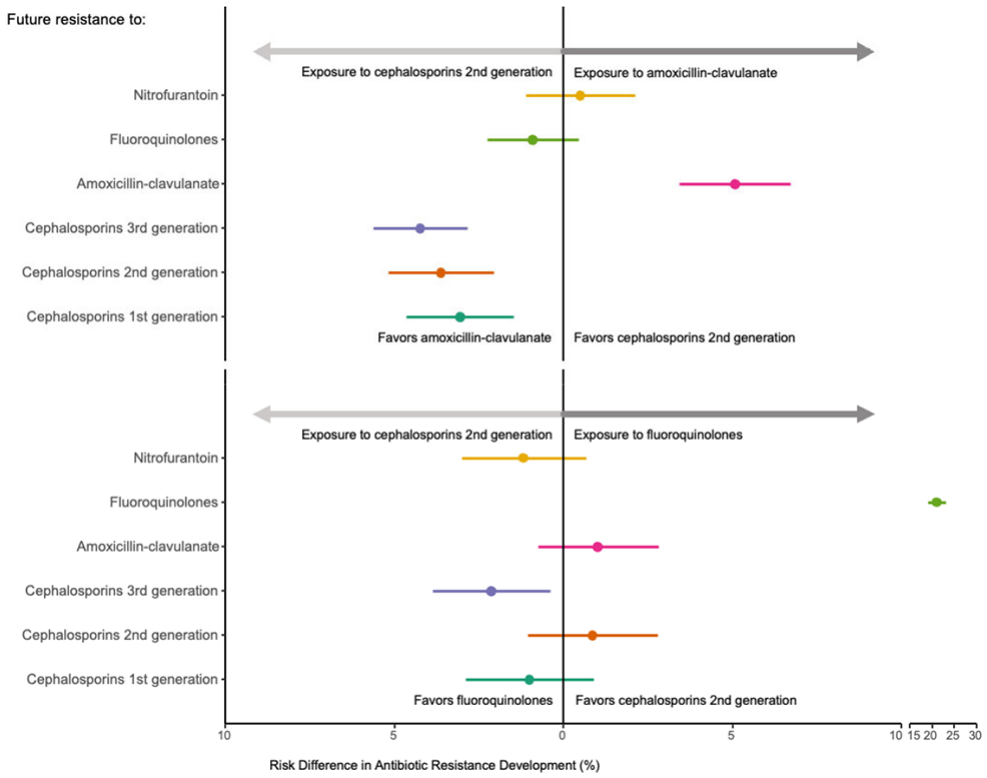
Risk Differences of Future Antibiotic Resistance When Exposed to Various Antibiotics Relative to 2nd CEP. The x-axis represents the risk difference in antibiotic resistance development, with horizontal lines depicting confidence intervals. The x-axis is split between 10–15 for visualization purposes. The y-axis depicts resistance to different antibiotics: nitrofurantoin, Ciprofloxacin, AMC, third-generation cephalosporins, 2nd CEP, and 1st CEP. The upper and lower panels represent comparisons with AMC and FQ, respectively. Positive risk differences indicate a higher risk of resistance development relative to 2nd CEP.

**Results:**

Included were 23,297 patients. Our analysis revealed that 1st CEPs were associated with the lowest resistance across all antibiotics. Exposure to any other antibiotic led to the highest resistance to itself. This effect was greatest when quinolones were examined as an exposure, RD 22.1%, 95%CI (20.0, 24.2) but was notable for AMC as well, RD 8.2%, 95%CI (6.1, 10.3). (figure 2) Second generation CEP induced higher resistance than AMC to all cephalosporins. However, as when compared with 1st CEP, resistance to AMC was highest under AMC exposure RD 5.1%, 95%CI(3.4, 6.7). Second generation CEP was associated with higher 3rd CEP resistance compared to FQ exposure, RD -2.1%, 95%CI (-3.9, -0.4). (Figure 3)

**Conclusion:**

Our study provides novel estimates of the resistance cost of different antibiotics. These estimates are relevant for both antibiotic treatment guidelines and clinical decision-making.

**Disclosures:**

All Authors: No reported disclosures

